# Efficacy of IP_6 _+ inositol in the treatment of breast cancer patients receiving chemotherapy: prospective, randomized, pilot clinical study

**DOI:** 10.1186/1756-9966-29-12

**Published:** 2010-02-12

**Authors:** Ivan Bačić, Nikica Družijanić, Robert Karlo, Ivan Škifić, Stjepan Jagić

**Affiliations:** 1 Department of Surgery, General Hospital Zadar, 23000 Zadar, Croatia; 2 Department of Surgery, University Hospital Split and University of Split School of Medicine, 21000 Split, Croatia; 3 Department of Statistics, University of Zadar, 23000 Zadar, Croatia

## Abstract

**Background:**

Prospective, randomized, pilot clinical study was conducted to evaluate the beneficial effects of inositol hexaphosphate (IP_6_) + Inositol in breast cancer patients treated with adjuvant therapy.

**Patients and methods:**

Patients with invasive ductal breast cancer where polychemotherapy was indicated were monitored in the period from 2005-2007. Fourteen patients in the same stage of ductal invasive breast cancer were involved in the study, divided in two randomized groups. One group was subjected to take IP_6 _+ Inositol while the other group was taking placebo. In both groups of patients the same laboratory parameters were monitored. When the treatment was finished, all patients have filled questionnaires QLQ C30 and QLQ-BR23 to determine the quality of life.

**Results:**

Patients receiving chemotherapy, along with IP_6 _+ Inositol did not have cytopenia, drop in leukocyte and platelet counts. Red blood cell counts and tumor markers were unaltered in both groups. However, patients who took IP_6 _+ Inositol had significantly better quality of life (p = 0.05) and functional status (p = 0.0003) and were able to perform their daily activities.

**Conclusion:**

IP_6 _+ Inositol as an adjunctive therapy is valuable help in ameliorating the side effects and preserving quality of life among the patients treated with chemotherapy.

## Introduction

Breast cancer is the most common cancer in women worldwide. Around 1.15 million cases were recorded in 2002, representing 23% of all female and 11% overall cancers [1]. Breast cancer incidence rates for 2002 vary internationally by more than 25-fold, ranging from 3.9 cases per 100 000 in Mozambique to 101.1 in the US, in part reflecting low screening rates and incomplete reporting in developing countries [2]. Breast cancer is fatal in almost half of all cases. It is the leading cause of cancer death from cancer among woman worldwide, accounting for 16% of cancer deaths in adult women [1,2].

Depending on the stage of breast cancer, the treatment is carried out by surgery, chemotherapy, ionizing radiation, hormone therapy and supportive measures that aim to reduce the side effects of treatment. Most patients are treated with chemotherapy in order to prevent the systemic dissemination of basic diseases. Patients are subjected to polychemotherapy - combination of three different drugs which are extremely aggressive and hard to bear. There are several protocols used in the treatment of breast cancer - FEC, FAC and CMF; FEC is the most frequently used protocol. Side effects of polychemotherapy (nausea, vomiting, loss of body weight, hair fall out, insomnia, depression, disorders in blood counts) appear in majority of patients and are the most common reasons for stopping the treatment. About 10% of patients interrupt the treatment which increases the risk of the appearance of distant metastasis, and reduces their chances for healing.

Inositol hexaphosphate (IP_6_) is a naturally occuring polyphosphorylated carbohydrate, present in almost all plant and mammalian cells, where it is important in regulating vital cellular functions such as signal transduction, cell proliferation and differentiation [3,4]. For a long time, IP_6 _has been recognized as a strong antioxidant. Recently, a striking anticancer effect of IP_6 _was demonstrated in different experimental models [3-14]. Inositol is also a natural constituent possesing moderate anticancer activity [3,4]. However, it was shown that inositol potentiates both the antiproliferative and antineoplastic effects of IP_6 _*in vivo*, and that the combination of IP_6 _and inositol was significantly better in different cancers (colon, breast and metastatic lung cancer model) than was either one alone [3,4]. Due to its strong antioxidant activity, and health beneficial effects, such as immune stimulation, prevention of kidney stone formation and hypocholesterolemic effect, IP_6 _+ Inositol is available as dietary supplement.

Current cancer treatment recognizes the importance of combination therapy in order to increase efficacy and decrease side effects of conventional chemotherapy. It has been shown *in vitro *that IP_6 _acts synergistically with doxorubicin and tamoxifen, being particularly effective against estrogen receptor-negative and doxorubicin-resistant breast cancer cell lines [15]. Furthermore, several case studies have shown that when IP_6 _and inositol were given in combination with chemotherapy, side effects of chemotherapy were diminished and patients were able to perform their daily activities [16-18]. Based on these properties, this study has been designed to evaluate in a small controlled clinical trial if the combination of IP_6 _+ Inositol and traditional chemotherapy will increase efficacy and decrease side effects of chemotherapy, and in particular if the IP_6 _+ Inositol will be able to improve the quality of life in patients undergoing the treatment for breast cancer.

## Materials and methods

### Study Population

In order to test the effectiveness of IP_6 _+ Inositol in improving the quality of life of patients who are treated for breast cancer, we have conducted a prospective, randomized, controlled clinical study with the tested (IP_6 _+ Inositol Group) and control (Placebo Group) groups of patients. This study was approved by the ethics committee of the General Hospital, Zadar. Written informed consent was obtained from all participants. The study included 14 patients with ductal invasive breast cancer subjected to surgery and with histological features and stage of tumor that indicated polychemotherapy. All patients received the FEC polychemotherapy protocol in six cycles. Patients receiving neoadjuvant chemotherapy were not included in the study.

Tested group consisted of 7 patients, average age 56 years (26-76), who were given IP_6 _+ Inositol (IP_6 _International Inc., Melbourne, FL, USA) per os in the form of powder in the daily dosage of 6 g, divided in 2 doses, starting from the first postoperative day, every day until the end of treatment (6 months). The control group consisted of 7 patients, who were treated for ductal invasive breast cancer of the same characteristics as the tested group. The control group was given placebo (Vitamin C) of the same look and consistency as IP_6 _+ Inositol, in the same dosage (6 g) until the end of treatment (6 months).

### Study Procedures

At the end of treatment, all patients have filled the questionnaires QLQ-30 and QLQ-BR23 from European organization for testing the treatment of cancer (EORTC) [19,20]. The questions in questionnaire were divided into two scales, the functional and symptomatic.

Functional scale contains questions about the physical, emotional, cognitive, social and sexual functions. Each group has a range of responses matching from 0-100, where 100 represents the maximum compatibility with the offered answers, and 0 represents the complete lack of compatibility.

Symptomatic scale contains questions about side effects of treatment, such as the general bad condition, nausea, vomiting, diarrhea, constipation, pain, insomnia, loss of appetite, loss of body weight, hair loss, increase body temperature and the operating complications of treatment. Replies from symptomatic scale are evaluated with the scale from 0-100, where 100 represents maximally positive personal experience with total quality of life, and 0 represents maximally negative personal experience of the quality of life.

Both groups of patients were monitored with the following laboratory parameters: total blood cell counts (TBC), CEA, CA 15-3, LDH, AST, ALT, AP, bilirubin, urea, creatinin and electrolytes. The testing was done at the first day of therapy, a month after, and at the the end of treatment.

For the processing of data obtained from the questionnaires, the QLQ-C30 SC (scoring manual), also produced by EORTC was utilized. The results were tested for significancy with the Student t-test for small samples (dependents and independents), and the p value of < 0.05 was considered significant.

## Results

All 14 patients involved in the study were regularly taking IP_6 _+ Inositol or placebo during 6 months. Not a single patient interrupted chemotherapy. The average age of life of patients who have taken IP_6 _+ Inositol was 56.2 years (26-76), while in the group of patients who had taken placebo average age of life was 59 years (42-77).

### The results of questionnaires about the quality of life (EORTC)

#### Personal assesment of quality of life

The average total personal experience with the quality of life was given in Table 1. Results of testing show that patients who have taken IP_6 _+ Inositol had statistically significantly higher quality of life than patients who were taking placebo (78.3 compared to 48.4; p = 0.05).

**Table 1:** Patients Personal Assessment of the Quality of Life

Quality of Life		
**Patients**	**Mean ± SD**	**p value**

Placebo Group	48.43 ± 28.96	0.05
	
IP_6 _+ Inositol Group	78.33 ± 21.60	

#### Functional scale (FS)

Answering questions about certain functions in everyday life, the average score was 87.9 in patients who have taken IP_6 _+ Inositol, while in patients who have taken placebo, the average score on the functional scale was 56.3 (p = 0.0003) (Table 2). The difference between the average scores between the two groups was statistically significant, showing that that the functional status of patients who were taking IP_6 _+ Inositol in addition to chemotherapy was significantly better preserved, in relation to the control group.

**Table 2:** Patients Personal Assessment of their Functional Status

Functional Status		
**Patients**	**Mean ± SD**	**p value**

Placebo Group	56.29 ± 15.32	0.0003
	
IP_6 _+ Inositol Group	87.94 ± 6.94	

#### Simptomatic scale (SS)

Among the patients who where taking IP_6 _+ Inositol, the average score of answers on questions about the symptomatic scale was 13.5, while that score in the control group was 33.8. The diference of the average scores between two groups is statistically significant (p = 0.04) (Table 3).

**Table 3:** Patients Personal Assessment of Side Effects of Therapy (Symptomatic Scale)

Clinical Symptoms of Side Effects of Therapy		
**Patients**	**Mean ± SD**	**p value**

Placebo Group	33.81 ± 18.12	0.04
	
IP_6 _+ Inositol Group	13.51 ± 9.98	

## Results of laboratory tests

Before treatment, the average number of leukocytes in the group of patients who were taking IP_6 _+ Inositol was 6.66 (5.1-7.7) × 10^9^/L, and after the treatment was 6.92 (3.8-9.1) × 10^9^/L, an average increase of 0.26. In the group of patients who were on placebo, the average number of leukocytes before treatment was 7.53 (6.2-10.4) × 10^9^/L and 4.36 (1,18-6.5) × 10^9^/L after the treatment; an average decrease of 3.17. In the control group of patients there was a statistically significant fall in the number of leukocytes after treatment compared to the number of leukocytes before treatment (p = 0.01), while in the experimental group on IP_6 _+ Inositol, not only that the number of leukocytes did not change (p = 0.75), but it was even slightly increased (Table 4).

**Table 4:** Change in Complete Blood Cell Count Values

Blood Cells		Placebo Group (Mean ± SD)	IP_6 _+ Inositol Group (Mean ± SD)
White Blood Cell Count (×10^9^/L)	Before Treatment	7.53 ± 1.50	6.66 ± 0.96
	
	After Treatment	4.36 ± 1.80	6.92 ± 2.12
	
	p value	0.01	0.75

Platelet Count (×10^9^/L)	Before Treatment	272.71 ± 114.86	229.57 ± 31.81
	
	After Treatment	205.00 ± 90.56	231.86 ± 47.33
	
	p value	0.05	0.92

Red Blood Cell Count (×10^12^/L)	Before Treatment	4.45 ± 0.71	4.23 ± 0.71
	
	After Treatment	4.05 ± 0.52	4.48 ± 0.23
	
	p value	0.23	0.39

Hemoglobin (g/L)	Before Treatment	122.00 ± 17.28	127.00 ± 19.94
	
	After Treatment	119.43 ± 10.78	135.86 ± 10.16
	
	p value	0.68	0.36

The average number of platelets before treatment was 229.57 (204-296) × 10^9^/L in a group of patients who were taking IP_6 _+ Inositol, while after the treatment it was 231.86 (182-322)× 10^9^/L, representing an increase of 2.29 × 10^9^/L platelets. In the control group, the average number of platelets before the treatment was 272.71 (176-525) × 10^9^/L, while after the treatment it was 205.00 (85-357) × 10^9^/L, representing a a drop of 67.71 × 10^9^/L (p = 0.05). Drop in the number of platelets in the control group of patients was statistically significant, while the number of platelets in the experimental group remained the same (Table 4).

In the IP_6 _+ Inositol group, the red bloood cell counts were 4.23 (3.56-5.22) × 10^12^/L and the hemoglobin level was 127.00 (110-151) g/L before treatment, while after the treatment the erythrocytes were 4.48 (4.08-4.78) × 10^12^/L and the hemoglobin level was 135.86 g L, representing an increase of 0.25 in the number of erythrocytes and 8.86 g/L in the hemoglobin level, although not significant. In the control group of patients the average number of erythrocytes before the treatment amounted to 4.45 × 10^12^/L, and 4.03 × 10^12^/L after the treatment, while the hemoglobin level prior to treatment was 122.00 (103-142) g/L and 119.43 (106-135) g/L after treatment, which represented a decrease of 0.4 in the average number of erythrocytes and decrease of 2.57 in the hemoglobin level. Changes in red blood cell counts and in the hemoglobin levels are not statistically significant for either group. These relations are evident from the Table 4.

There were no significant changes in tumor markers CEA and CA 15-3 during the treatment in both groups. For CEA, preoperative average value in the IP_6 _+ Inositol group was 3.01 ng/mL (1.0-6.7), and postoperative value was 3.15 ng/mlL (1.5-6.9), which amounted to a nonsignificant average increase of 0.14 ng/mL (p = 0.39). In the control group of patients, preoperative average value for CEA was 2.40 ng/mL (1.2-5.3), while the postoperative average CEA value was 2.48 ng/mL, representing an average increase of 0.08 ng/mL (p = 0.87) (Table 5). Preoperative average value of CA 15-3 in the IP_6 _+ Inositol group was 13.05 U/mL (9.2-16.3), postoperative 13.80 U/mL (10.3-17.2), which was an increase of 0.75 U/mL (p = 0.08). In the control group, the average preoperative value for CA 15-3 was 26.27 U/mL ((12.7-49.6) and postoperative value was 27.41 U/ml (11.9-62), representing an increase of 1.14 U/mL (p = 0.86) (Table 5).

**Table 5:** Values of Tumor Markers CEA and CA15-3

Tumor Markers		Placebo Group (Mean ± SD)	IP_6 _+ Inositol Group (Mean ± SD)
CEA (ng/mL)	Before Treatment	2.40 ± 1.53	3.01 ± 1.80
	
	After Treatment	2.48 ± 1.27	3.15 ± 1.85
	
	p value	0.87	0.39

CA 15-3 (kU/L)	Before Treatment	26.27 ± 15.20	13.05 ± 2.35
	
	After Treatment	27.41 ± 17.28	13.80 ± 2.67
	
	p value	0.86	0.08

Other laboratory parameters that were monitored during the treatment (LDH, AST, ALT, AP, bilirubin, urea, creatinine, and electrolytes) were stable in both groups of patients and there were no deviations from the reference value.

## Discussion

IP_6 _has been known for a long time for its beneficial effects, including boosting immune system, preventing kidney stone formation and lowering serum cholesterol [3,4]. Additionally, IP_6 _has shown a significant anticancer effect against different experimental cancers [3-15]. For some time, IP_6 _is available as a dietary supplement. Although few case studies in which IP_6 _plus inositol was given in combination with chemotherapy clearly showed encouraging data, organized, controlled, randomized clinical studies were never organized [16-18]. Therefore, this study conducted at the Department of Surgery, General Hospital, Zadar on the group of voluntary patients who were treated for breast cancer, is the first study of its kind in the world. From this small clinical testing we concluded that IP_6 _+ Inositol was able to improve the quality of life of breast cancer patients undergoing chemotherapy compared to control, placebo group with the same histological type of cancer and the therapeutic protocol.

It is difficult to be objective and to numerically express the quality of life of individual patients or groups of patients in order to compare the quality of life of another patient, because it depends on a number of parameters. The European Association for research and treatment of cancer (EORTC) has developed questionnaires for assessing the quality of life of patients which have fallen ill from cancer, and thus tried to compare objectively the quality of life that we utilized. Our results show that patients who were taking IP_6 _+ Inositol in combination with chemotherapy, had overall statistically significantly better quality of life than patients who were on placebo. Analyzing the answers to questions about the side effects of treatment and symptoms of disease, we have seen that the frequency and intensity of side effects associated with patients who were taking IP_6 _+ Inositol were statistically significantly lower in comparison to patients who were taking placebo.

Drugs that are implemented in chemotherapy are agressive and have impact to the tumor cells as well as to the cells in the blood. Most patients who are undergoing chemotherapy have some anomalies in their complete blood count, primarily in the number of leukocytes and plateletes. Our results show that patients who have taken IP_6 _+ Inositol did not show drop in the number of leukocytes and plateletes, on the contrary, these were even slightly increased. A slight increase in red blood cell counts and hemoglobin levels were also noticed in the IP_6 _+ Inositol group. Tumor markers, liver enzymes, bilirubin, urea, creatinine and electrolytes were not disturbed in either group during the 6-month period of treatment.

Although our clinical study was conducted on a small number of patients, our results confirmed previous observations and clearly demonstrated that IP_6 _+ Inositol when included in chemotherapy for breast cancer significantly improved patients' quality of life and protected patients from the loss in the number of leukocytes and plateletes [16-18].

The question remains to what extent we can include IP_6 _+ Inositol in the therapeutic program for breast cancer. The dosage of 6 g daily represents a low dose level of IP_6 _+ Inositol. Extrapolated from animal data, in the absence of a dose-determination study in humans, the recommended prophylactic dosage of IP_6 _+ Inositol is 1-2 g/day and a cancer therapeutic dosage is 8-12 g/day [4]. Even though our dosage was low, its efficacy to diminish the side effects of chemotherapy was significant.

Recent phase I study of inositol for lung cancer chemoprevention showed that in a daily dose of 18 g p.o. for 3 months, inositol was safe and well tolerated [21]. Recently it was reported that the combination of beta-(1,3)/(1,6) D-glucan and IP6 was well tolerated and had beneficial effect on hematopoesis in the treatment of patients with advanced malignancies receiving chemotherapy [22]. Although the results of our pilot studies are encouraging, it is necessary to conduct further multicentric clinical testing on a larger number of patients for further evaluation of the impact that IP_6 _+ Inositol on the quality of life of patients treated from breast cancer.

## Competing interests

The authors declare that they have no competing interests.

## Authors' contributions

IB formulated the research protocol and carried out the follow up of participants. ND and SJ participated in the design of the study and performed the statistical analysis. RK and IS participated in the design of the study, and the execution of the study protocol. All authors read and approved the final manuscript.
